# Comprehensive Analysis of Expression and Prognostic Value of Sirtuins in Ovarian Cancer

**DOI:** 10.3389/fgene.2019.00879

**Published:** 2019-09-13

**Authors:** Xiaodan Sun, Shouhan Wang, Qingchang Li

**Affiliations:** ^1^Department of Pathology, College of Basic Medical Sciences, China Medical University, Shenyang, China; ^2^Department of 2^nd^ Gynecologic Oncology Surgery, Jilin Cancer Hospital, Changchun, China; ^3^Department of Hepatopancreatobiliary Surgery, Jilin Cancer Hospital, Changchun, China; ^4^Department of Pathology, the First Affiliated Hospital, China Medical University, Shenyang, China

**Keywords:** sirtuins, ovarian cancer, prognosis, database, bioinformatics analysis

## Abstract

Sirtuins (SIRTs) 1–7 are a family of intracellular enzymes, which possess nicotinamide adenine dinucleotide-dependent deacetylase activity. Emerging evidence suggest that SIRTs play vital roles in tumorigenesis by regulating energy metabolism, DNA damage repair, genome stability, and other cancer-associated cellular processes. However, the distinct roles of the seven members in ovarian cancer (OC) remain elusive. The transcriptional expression patterns, prognostic values, and genetic alterations of seven SIRTs in OC patients were investigated in this study using a range of databases: Oncomine and Gene Expression Profiling Interactive Analysis, Kaplan–Meier plotter, the Cancer Genome Atlas, and cBioPortal. The protein–protein interaction networks of SIRTs were assessed in the String database. Gene Ontology enrichment and Kyoto Encyclopedia of Genes and Genomes pathway were analyzed in Database for Annotation, Visualization, and Integrated Discovery. The mRNA expression levels of SIRT1–4 and 7 were downregulated, while that of SIRT5 was upregulated and SIRT6 exhibited both expression dysregulation in patients with OC. Dysregulated SIRTs mRNA expression levels were associated with prognosis. Moreover, genetic alterations primarily occurred in SIRT2, 5, and 7. Network analysis indicated that SIRTs and their 20 interactors were associated with tumor-related pathways. This comprehensive bioinformatics analysis revealed that SIRT1–4, 6, and 7 may be new prognostic biomarkers, while SIRT5 is a potential target for accurate therapy for patients with OC, but further studies are needed to confirm this notion. These findings will contribute to a better understanding of the distinct roles of SIRTs in OC.

## Introduction

Ovarian cancer (OC) ranked eighth in incidence and seventh in mortality rates globally among all cancers in women in 2018 (WHO, http://gco.iarc.fr/today/home). Furthermore, the absence of incipient symptoms leads to over three quarters of patients being diagnosed at advanced stages (Zhou et al., 2018). Standard treatment for this disease involves surgical intervention combined with chemotherapy. Although the use of gene sequencing and targeted therapies have improved the survival of OC patients, the 5-year survival rate is still poor because of the complex tumor processes and pathological subtypes of OC and the shortage of more specific target biomarkers. Therefore, enhancing therapy requires new biomarkers for prognosis and individualized treatment of OC.

Sirtuins (SIRTs) are a family of intracellular enzymes that possess nicotinamide adenine dinucleotide (NAD^+^)-dependent deacetylase activity and share a highly conserved 275-amino catalytic core domain. Seven members (SIRT1–7) in mammals are divided into the following four classes: SIRT1–3, I; SIRT4, II; SIRT5, III; and SIRT6-7, IV (O’Callaghan and Vassilopoulos, 2017). Based on their subcellular localization, they can also be categorized as follows: SIRT1, 6, and 7 reside in the nucleus; SIRT2 is expressed in both the nucleus and cytoplasm; and SIRT3, 4, and 5 are in the mitochondria (Chalkiadaki and Guarente, 2015). Emerging evidence suggest that SIRTs play vital roles in tumorigenesis by regulating energy metabolism, DNA damage repair, genome stability, and various other cancer-associated cellular processes. Aberrant expression of SIRTs has been found in common human carcinomas such as breast, lung, liver, and gastrointestinal cancers, as well as OC and neurologic tumors (Chen et al., 2013; Chalkiadaki and Guarente, 2015; Osborne et al., 2016; O’Callaghan and Vassilopoulos, 2017).

Presently, the dysregulated expression of SIRTs and their prognostic value have been partly reported in OC. For example, the expression of SIRT1 was found to be higher in 68 OC tissue samples than it was in 16 normal ovaries (Mvunta et al., 2017). Consistent with this study, overexpression of SIRT1 was also reported in 90 OC tissue samples compared with 40 normal ovary tissues, and, interestingly, a high expression level of SIRT1 was associated with a favorable outcome (Jang et al., 2009). However, a converse finding that SIRT1 was downregulated in OC based on public datasets has also been reported (Hyde et al., 2018). SIRT2 predicted poor survival when upregulated in patients with OC (Teng and Zheng, 2017), while reduced expression of SIRT2 was observed in 13 samples of serous ovarian carcinoma compared with 11 samples of normal ovarian surface epithelial tissues (Du et al., 2017). At least one copy of the *SIRT3* gene was deleted in 40% of breast and OCs, and focal deletions of *SIRT3* were especially frequent in ovarian tumors (Finley et al., 2011). In contrast, the region encompassing the *SIRT5* locus was amplified in 30% of high-grade serous ovarian carcinomas (Bell et al., 2011a). SIRT3 and SIRT5 expression were found to be significantly decreased and increased in primary serous OCs/tubal cancers compared with that in normal counterparts, respectively (Li et al., 2019). SIRT4 has been reported to function as a tumor suppressor in published studies, and reduced expression in OC was reported in a meta-analysis (Csibi et al., 2013).The mRNA expression of SIRT6 in 32 OC tissue samples was remarkably lower than that in paired normal ovarian tissues (Zhang et al., 2015), whereas there were higher SIRT7 mRNA levels in OC, although without statistical significant, which could have been due to the small sample sizes analyzed (Aljada et al., 2015).

These findings indicate that SIRTs are closely associated with OC, and it is striking that even in the same tumor, the specific roles of individual SIRTs can be controversial, which may be partly ascribed to small sample sizes. A comprehensive analysis of the expression and mutation patterns and prognostic values of SIRTs in OC based on large database analysis would enhance the understanding of their potential roles in OC. Therefore, we conducted this study to investigate this phenomenon.

## Methods

### Ethics Statement

The OC specimens and normal tissues were obtained from patients who were diagnosed with OC and underwent primary cytoreductive (debulking) surgery from Aug 2017 and May 2018 in First Affiliated Hospital, China Medical University. The enrolled patients had signed informed consent. This study was approved by the Medical Research Ethics Committee of China Medical University and conducted according to the principles expressed in the Declaration of Helsinki. All the datasets were retrieved from the published literature, so it was confirmed that all written informed consent was obtained.

### Oncomine Database

The Oncomine database (www.oncomine.org) (Rhodes et al., 2004), an online cancer microarray database and web-based data-mining platform, was used to investigate the transcriptional levels of SIRTs in different clinical cancer specimens and corresponding normal controls. The search contents and thresholds were set as follows: keywords, SIRT1–SIRT7, primary filter, cancer vs. normal; cancer type, OC, the absolute value of log_2_ fold change >1.5, *P* < 0.05; and gene rank, 10%. The *P* value was calculated using the Student’s *t* test.

### GEPIA Database

The Gene Expression Profiling Interactive Analysis (GEPIA) database (http://gepia.cancer-pku.cn/), a newly developed web-based tool, provides key interactive and customizable functions including tumor vs. normal differential expression analysis, profiling plotting in accordance with cancer types or different pathological stages, correlation analysis, patient survival analysis, similar gene detection, and dimensionality reduction analysis based on the Cancer Genome Atlas (TCGA) and the genotype–tissue expression data (Tang et al., 2017).

### The Kaplan–Meier Plotter

The prognostic value of SIRTs in OC patients was evaluated using the Kaplan–Meier plotter (http://kmplot.com/analysis), an open online dataset that can be used to assess the effect of 54,675 genes on survival in 21 cancer types including breast, liver, ovarian, lung, and gastric cancer (Győrffy et al., 2012). To analyze the overall survival (OS) and progression-free survival (PFS) of patients with OC, samples were split into two groups based on median expression (high vs. low). The hazard ratio (HR) with 95% confidence intervals (CIs) and log-rank *P* values were calculated and displayed in survival plots. *P* < 0.05 was considered statistically significant.

### TCGA Database and cBioPortal

The cBioPortal for Cancer Genomics (http://cbioportal.org) provides an open-access web resource for exploring, visualizing, and analyzing multidimensional cancer genomic data from TCGA (Gao et al., 2013). In the present study, three TCGA datasets of OC, namely, “TCGA Nature 2011 (563 cases),” “TCGA PanCancer Atlas (585 cases),” and “TCGA Provisional (606 cases)” were selected for further analysis of *SIRT* gene mutations or copy number alterations (CNA). The OncoPrint, survival tabs were applied according to the online instructions of the cBioPortal.

### String Database and DAVID

The interaction proteins network of SIRTs was constructed using the String Database (https://string-db.org/), which is an online database of predicted functional associations between proteins (von Mering et al., 2003). “*Homo sapiens*” was selected and interactions with a combined score >0.7 (high confidence) were considered significant. Seven SIRTs and 20 associate proteins were imported into Database for Annotation, Visualization, and Integrated Discovery (DAVID) (https://david.ncifcrf.gov/) to perform Gene Ontology (GO) and Kyoto Encyclopedia of Genes and Genomes (KEGG) analyses (Huang et al., 2009a; Huang et al., 2009b). The human genome was selected as the background parameter, and a *P* < 0.05 was considered statistically significant.

### Immunohistochemistry

Surgically excised normal and tumor specimens were fixed in 10% neutral formalin, embedded in paraffin, and cut into 4-mm sections. The sections were incubated with commercial rabbit polyclonal antibodies against SIRT1, SIRT2, SIRT3, SIRT4, SIRT5, SIRT6, and SIRT7 (SIRT1, 2, 5–7 were purchased from Proteintech, China; SIRT3 and SIRT4 were purchased from Abcam, China) at 1/100 dilution overnight at 4°C. Then, the reaction was visualized using the Elivision super HRP IHC Kit (Maixin-Bio) and 3,3-diaminobenzidine (DAB); nuclei were counterstained with hematoxylin. The sections were dehydrated in ethanol before mounting.

### Cell Culture and Quantitative Real-Time PCR Analysis

The A2780 and SKOV-3 human OC cell lines were used in this study. The cells were cultured in Dulbecco’s modified Eagle medium and RPMI-1640, respectively, supplemented with 10% fetal bovine serum. These cells were grown at 37°C in a humidified atmosphere with 5% CO_2_.

Trizol (Invitrogen, Carlsbad, CA) was used to extract total RNA from OC cells. One microgram RNA was reverse transcripted using the PrimeScript RT Master Mix (TaKaRa) according to manufacturer’s instructions. Quantitative real-time PCR (qRT-PCR) was done using Applied Biosystems Power SYBR Green on a qTOWER2.0. Real-time PCR system is as follows: 10 s at 95°C, then 40 cycles at 95°C for 5 s, and 65°C for 34 s. The gene amplification specificity was shown by a melting curve generated in dissociation procedure. 2^−ΔΔCt^ method was used to normalize the quantification of SIRT1-7 to glyceraldehyde 3-phosphate dehydrogenase (GAPDH). The specific primer sequences are performed as follows:

**Table:** 

GAPDH Forward 5′-CCACCCATGGCAAATTCC-3′	Reverse 5′-GATGGGATTTCCATTGATGACA-3′
SIRT1 Forward 5′-GTAGGCGGCTTGATGGTAATC-3′	Reverse 5′-GACTCTGGCATGTCCCACTAT-3′
SIRT2 Forward 5′-GCGGAACTTATTCTCCCAGAC-3′	Reverse 5′-GCTCCCACCAAACAGATGAC-3′
SIRT3 Forward 5′-CTGTGGGTGCTTCAAGTGTTG-3′	Reverse 5′-CCCGAATCAGCTCAGCTACAT-3′
SIRT4 Forward 5′- ACACTGGGCTTTGAGCACCT-3′	Reverse 5′-GAGTCTGTTCCCCACAATCCA -3′
SIRT5 Forward 5′-TCGTGGTCATCACCCAGAAC-3′	Reverse 5′-GCCACAACTCCACAAGAGGTAC-3′
SIRT6 Forward 5′-GCCAAGTGTAAGACGCAGTAC-3′	Reverse 5′-TAGGATGGTGTCCCTCAGCT-3′
SIRT7 Forward 5′-CATCGTGAACCTGCAGTGGA-3′	Reverse 5′-GGGAGTCGCCAGTGAGAAAA-3′

## Results

### Transcriptional Levels of SIRTs and Their Relationship With Clinicopathological Characters in Patients With OC

**Figure 1:**
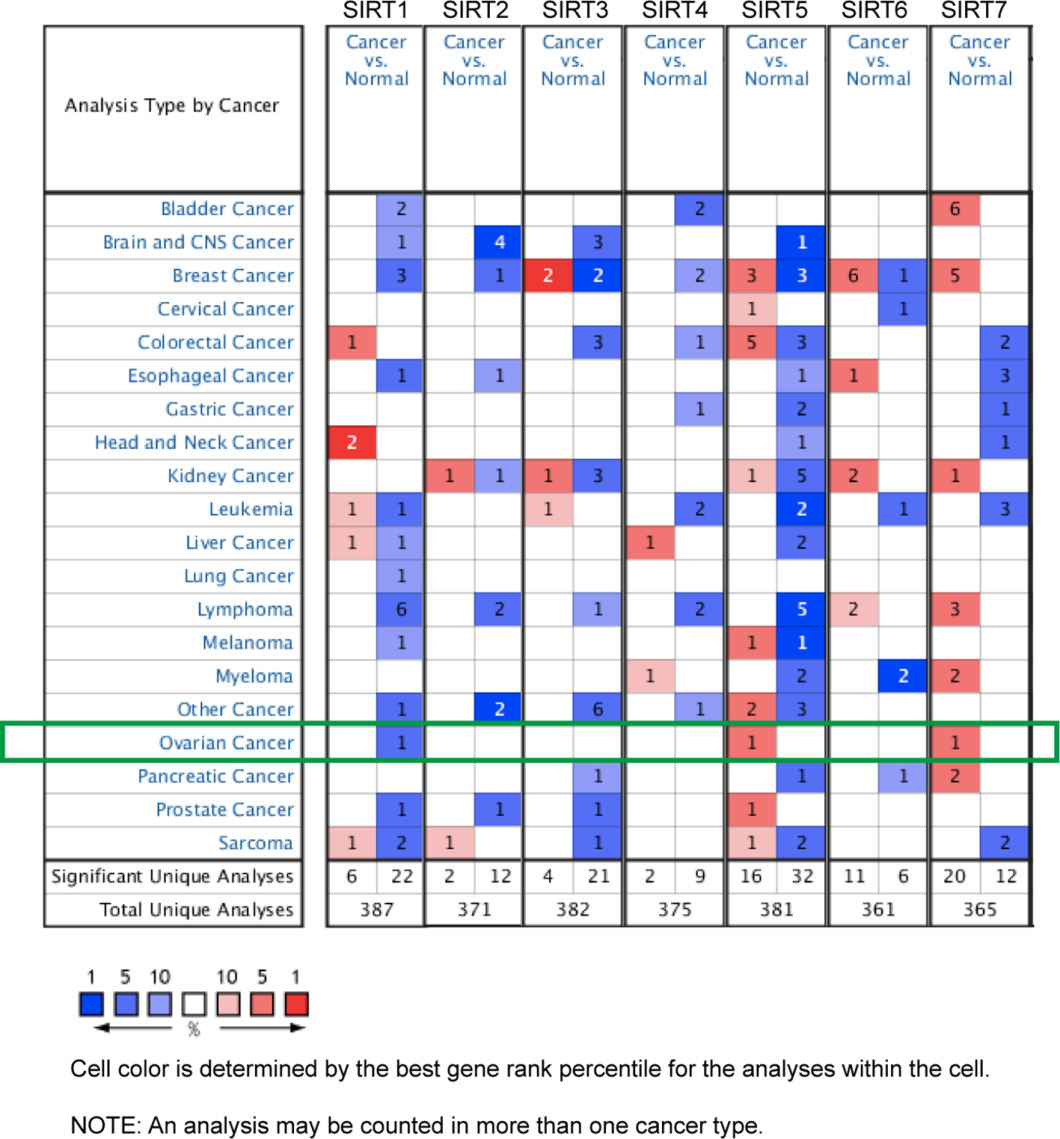
The mRNA levels of sirtuins (SIRTs) in 20 different types of cancers (Oncomine). The number in each cell represents the number of analyses that satisfied the following threshold: *P* < 0.05, the absolute value of log_2_ fold change >1.5, and gene rank, 10%. The numbers in colored cells show the quantities of datasets with statistically significant mRNA overexpression (red) or downexpression (blue) of target genes.

The dysregulated transcriptional levels of seven SIRTs have been identified in 20 different types of human cancers in the Oncomine database. As shown in **Figure 1**, SIRTs might act as either a tumor promoter or suppressor, in a context-specific manner. Especially, the mRNA expression levels of SIRT1 were significantly downregulated in patients with OC in Bonome’s dataset (Bonome et al., 2008) with a log_2_ fold change of −1.866, while SIRT5 and SIRT7 were higher in ovarian serous adenocarcinoma in two another datasets (Yoshihara Ovarian and TCGA datasets; log_2_ fold changes, 1.929 and 1.626, respectively) (Yoshihara et al., 2009) than in normal ovarian tissues (**Table 1**, bold font).

**Table 1:** The significant changes of sirtuin (SIRT) expression between different types of OC and normal tissues (Oncomine).

Sirtuins	Types of OC vs. Normal															Ref/Source
	Ovarian Carcinoma			Serous			Endometrioid			Mucinous			Clear cell			
	Log_2_ FC	*P*	N	Log_2_ FC	*P*	N	Log_2_ FC	*P*	N	Log_2_ FC	*P*	N	Log_2_ FC	*P*	N	
SIRT1	**−1.866**	**1.19E-9**	185	–	–	–	–	–	–	–	–	–	–	–	–	Bonome ovarian
	–	–	–	−1.172	1.67E-5	41	−1.142	4.92E−5	37	−1.174	1.62E−5	13	−1.128	0.001	8	Hendrix ovarian
SIRT2	–	–	–	−1.146	0.021	20	−1.179	0.011	9	NS	NS	9	NS	NS	7	Lu ovarian
SIRT3	–	–	–	−1.151	0.004	41	−1.150	0.005	37	−1.159	0.013	13	−1.153	0.012	8	Hendrix ovarian
SIRT4	–	–	–	−1.177	3.30E-6	41	−1.171	4.82E−6	37	−1.179	9.29E−4	13	−1.074	0.049	8	Hendrix ovarian
SIRT5	–	–	–	1.115	1.29E-4	41	1.041	0.033	37	NS	NS	13	NS	NS	8	Hendrix ovarian
	–	–	–	**1.929**	**6.44E-7**	43	–	–	–	–	–	–	–	–	–	Yoshihara ovarian
SIRT6	–	–	–	NS	NS	41	1.033	0.007	37	1.056	5.15E−4	13	1.070	0.002	8	Hendrix ovarian
SIRT7	−1.097	0.025	185	–	–	–	–	–	–	–	–	–	–	–	–	Bonome ovarian
	–	–	–	**1.626**	**1.71E-8**	586	–	–	–	–	–	–	–	–	–	TCGA
	–	–	–	1.163	1.95E-8	41	1.15	8.14E−8	37	1.255	1.45E−4	13	1.233	1.33E−4	8	Hendrix ovarian

The bold font indicates the difference between OC and normal tissues meets the selected thresholds.

OC, ovarian cancer; FC, fold change; NS, not significant; “–”, not available; N, number of patients.

Moreover, the mRNA levels of SIRTs in different types of OC, which were available in Oncomine datasets, are summarized in **Table 1**. In Hendrix’s dataset, SIRT1, SIRT3, and SIRT4 expression levels were significantly lower in serous, endometrioid, mucinous, and clear cell adenocarcinoma than they were in normal ovarian tissues. SIRT2 expression was lower in serous and endometrioid adenocarcinoma in Lu’s dataset (Lu et al., 2004), whereas SIRT5 was upregulated in those types of OC in Hendrix’s dataset compared with normal tissues (Hendrix et al., 2006). SIRT6 was expressed at higher levels in all types of OC than it was in normal tissues in Hendrix’s dataset except for serous adenocarcinoma. Interestingly, SIRT7 was downregulated in OC in Bonome’s dataset but upregulated in both TCGA and Hendrix’s datasets compared with normal tissues (Hendrix et al., 2006; Bonome et al., 2008).

**Figure 2:**
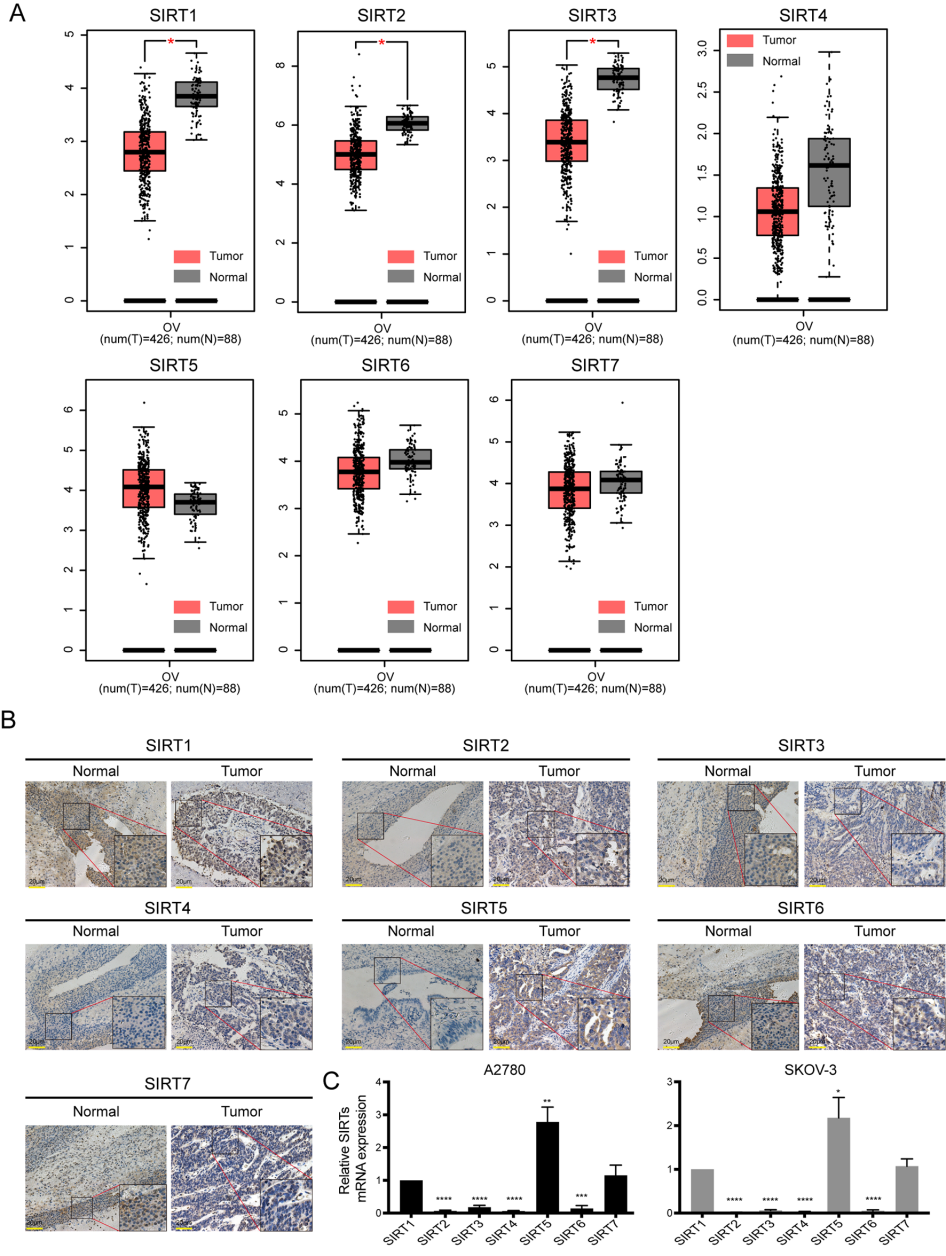
The mRNA and protein expression of SIRTs in patients with ovarian cancer (OC). **(A)** Box plots of SIRTs mRNA expression based on GEPIA database. **(B)** The representative immunohistochemical staining images of SIRTs protein expression in ovarian cancer and normal tissues (magnification, ×400; scale bar = 20 µm). **(C)** The mRNA levels of SIRTs in A2780 and SKOV-3 ovarian cell lines by quantitative real-time PCR (qRT-PCR).*P < 0.05, **P < 0.01,***P < 0.001, ****P < 0.00001.

**Figure 3:**
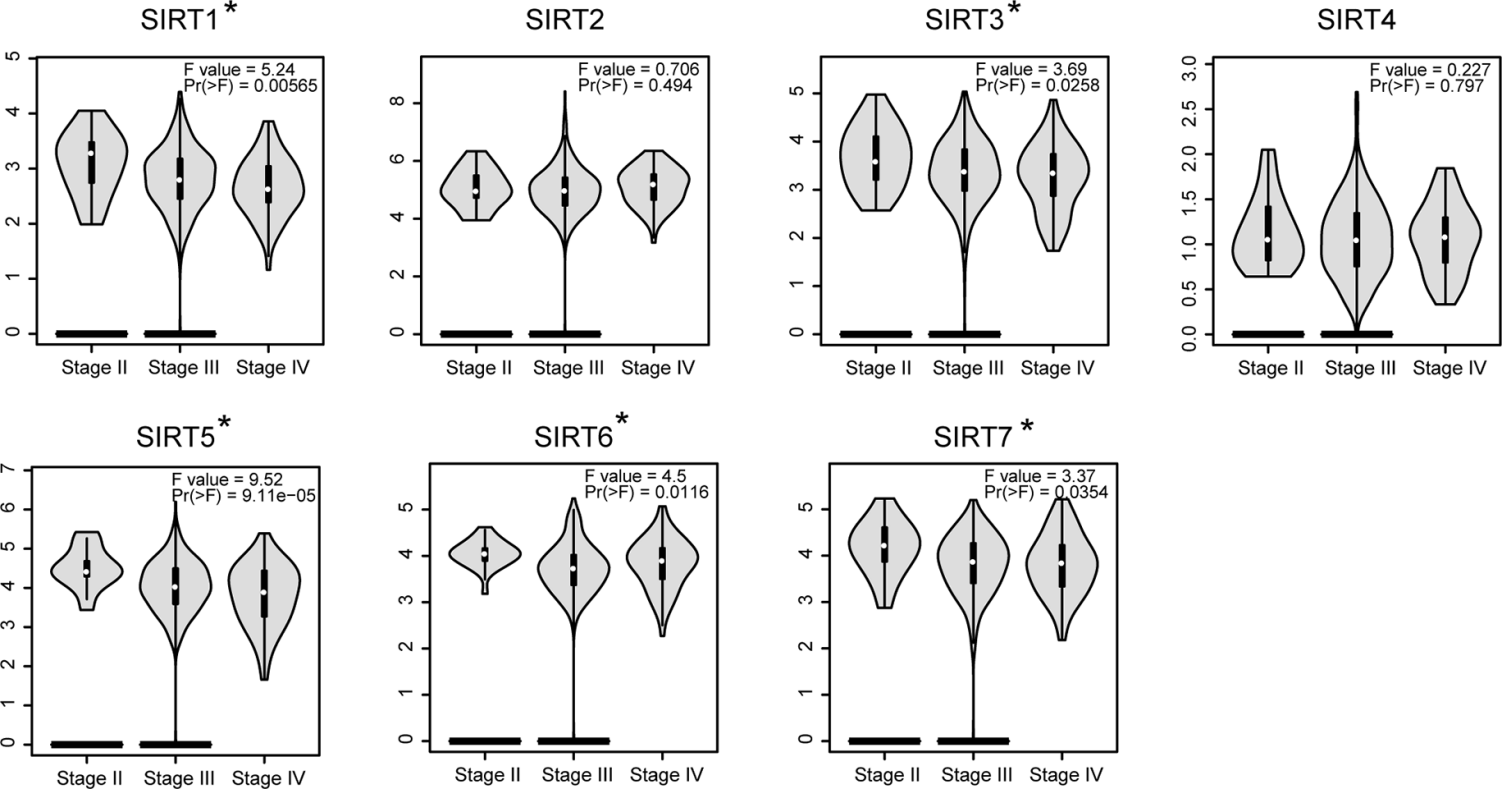
The relationship between SIRTs mRNA expression and tumor stages in patients with OC [Gene Expression Profiling Interactive Analysis (GEPIA)]. *P < 0.05.

In addition, the GEPIA database was also used to compare the mRNA expression of SIRTs between OC and normal tissues. The expression levels of SIRT1–3 were significantly lower, and levels of SIRT4, 6, and 7 were slightly more downregulated (*P* > 0.05) in OC than they were in normal tissues, while SIRT5 exhibited contrasting expression (**Figure 2A**). The results were consistent with those of the Oncomine database except for that of SIRT6. These findings were verified by immunochemistry (IHC), and as shown in **Figure 2B**, SIRT5 protein expression was higher in OC than in the counterpart normal tissues, while the protein expression difference of other SIRTs was not significant. Furthermore, the mRNA levels of SIRTs in two OC cell lines were detected by qRT-PCR, and the results were similar to the IHC (**Figure 2C**). The relationship between mRNA expression levels of SIRTs and different tumor stages of OC were also analyzed, and they were all significantly upregulated in stage II except for SIRT2 and SIRT4 (**Figure 3**).

### Prognostic Value of SIRTs in Patients With OC

**Figure 4:**
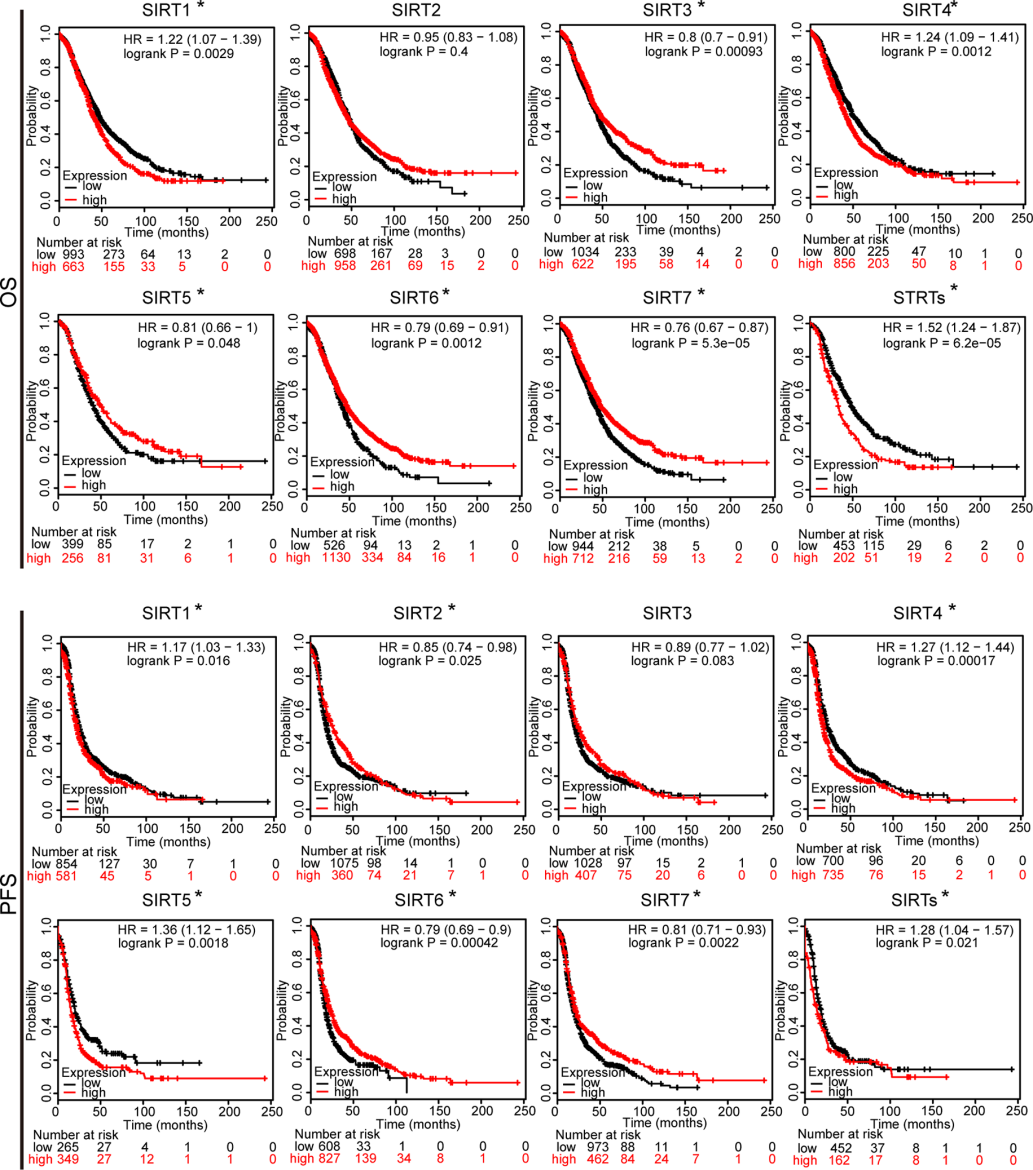
The prognostic value of mRNA level of SIRTs in patients with OC (Kaplan–Meier plotter). *P < 0.05.

To further assess the prognostic value of SIRTs in all patients with OC, Kaplan–Meier plotter analysis was used. We initially assessed the relationship between the mRNA expression of individual SIRT and the survival of OC patients. The survival curves demonstrated that decreased SIRT1 and SIRT4 mRNA levels and increased expression of SIRT2, 3, 6, and 7 predicted favorable prognosis (OS and PFS). Interestingly, a higher level of SIRT5 was associated with shorter PFS but with longer OS. Then, we also wondered the prognostic value of the combined SIRTs, and the results showed that upregulated levels of their combined mRNA expression was correlated with poor outcome in patients with OC (**Figure 4**).

Moreover, we also assessed the prognostic values of SIRTs in different subtypes of OC, namely, different histology, clinical stages, pathological grades, and TP53 status, which are available in Kaplan–Meier plotter. As shown in **Table 2**, increased mRNA expression of SIRT3, 5, 6, and 7 in serous OC patients and decreased levels of SIRT4 in both serous and endometrioid OC patients were significantly related to improved OS. The overexpression of SIRT2–4 predicted shorter PFS in serous OC patients. As shown in **Table 3**, high mRNA expression of SIRT5 and low expression of SIRT6, 7 were associated with poor OS in stage 1. Elevated mRNA levels of SIRT3, 5–7 and low levels of SIRT1, 4 were associated with better OS in stage 3, while high level of SIRT2 predicted poor OS in stage 4. In terms of pathological grades, high SIRT6 mRNA expression was linked to favorable OS. Interestingly, increased expression of SIRT3 predicted poor OS in mutated TP53 type, while it was associated with better OS in wild-type TP53. With respect to PFS (**Table 4**), high mRNA expression of SIRT1-3 and 7 were found to be correlated to shorter PFS in stage 1, whereas low levels of SIRT1, 5 and SIRT2, 4, and 6 predicted longer PFS in stages 2 and 3, respectively. In stage 4, increased expression of SIRT2 and 3 were linked to poor PFS. With regard to pathological grades, decreased levels of SIRT2 and 4 predicted better PFS. Interestingly, SIRT3 exhibited opposite roles in different pathological grades. Additionally, elevated expression of SIRT1 and 2 were associated with poor PFS in both mutated and wild type of TP53, while increased levels of SIRT3, 6, and 7 were related to poor PFS in mutated TP53 status. Taken together, these results indicated that the mRNA expression levels of SIRTs may be potential biomarkers for the prediction of OC patient survival.

**Table 2:** The prognostic values of SIRTs in different pathological subtypes OC (Kaplan–Meier plotter).

Sirtuins	Histology	OS			PFS		
		Cases	HR(95% CI)	*P* value	Cases	HR(95% CI)	*P* value
SIRT1 218878_s_at	Serous	1,207	1.15(0.99–1.34)	0.074	1,104	0.88(0.75–1.03)	0.1
	Endometrioid	37	4.94(0.82–29.69)	0.053	51	0.56(0.22–1.43)	0.22
SIRT2 220605_s_at	Serous	1,207	1.13(0.95–1.33)	0.17	1,104	1.4(1.2–1.63)	**1.6E−05**
	Endometrioid	37	3.84(0.43–34.41)	0.10	51	2.08(0.82–5.27)	0.11
SIRT3 221913_at	Serous	1,207	0.82(0.7–0.95)	**0.0096**	1104	1.21(1.03–1.41)	**0.019**
	Endometrioid	37	0.46(0.08–2.75)	0.38	51	4.92(0.65–36.99)	0.086
SIRT4 220047_at	Serous	1,207	1.22(1.05–1.42)	**0.011**	1104	1.26(1.09–1.45)	**0.0019**
	Endometrioid	37	9.36(1.04–84.6)	**0.016**	51	0.64(0.21–1.94)	0.42
SIRT5 229112_at	Serous	523	0.78(0.62–0.98)	**0.036**	483	1.17(0.94–1.47)	0.17
	Endometrioid	30	3.01(0.31–29)	0.32	44	1.51(0.47–4.83)	0.48
SIRT6 219613_s_at	Serous	1,207	0.81(0.69–0.94)	**0.0062**	1104	1.14(0.97–1.33)	0.11
	Endometrioid	37	0.17(0.02–1.5)	0.069	51	1.97(0.7–5.55)	0.19
SIRT7 218797_s_at	Serous	1,207	0.8(0.69–0.93)	**0.0044**	1104	1.1(0.93–1.3)	0.28
	Endometrioid	37	–	0.18	51	1.99(0.79–5.03)	0.14

The bold font indicates the difference was significant statistically. “–”, not available;

OC, ovarian cancer; OS, overall survival; PFS, progression-free survival.

**Table 3:** The relationship between SIRTs and OS in other different subtypes of OC (Kaplan–Meier plotter).

			SIRT1		SIRT2		SIRT3		SIRT4		SIRT5		SIRT6		SIRT7	
Subtypes		Cases	HR(95% CI)	*P*	HR(95% CI)	*P*	HR(95% CI)	*P*	HR(95% CI)	*P*	HR(95% CI)	*P*	HR(95% CI)	*P*	HR(95% CI)	*P*
Stage	1	74	2.38 (0.75–7.54)	0.13	0.34 (0.11–1.08)	0.056	2.31 (0.62–8.55)	0.2	0.51 (0.14–1.88)	0.3	5.65 (1.13–28.18)	**0.017**	0.31 (0.1–0.96)	**0.033**	0.28 (0.09–0.88)	0.02
	2	61	1.68 (0.56–5.06)	0.35	0.64 (0.21–1.9)	0.42	0.55 (0.18–1.66)	0.28	0.38 (0.12–1.18)	0.082	0.27 (0.05–1.44)	0.1	0.3 (0.07–1.36)	0.099	0.52 (0.18–1.51)	0.22
	3	1044	1.21 (1.03–1.42)	**0.024**	0.92 (0.77–1.09)	0.33	0.75 (0.63–0.88)	**0.0005**	1.29 (1.08–1.54)	**0.005**	0.7 (0.55–0.91)	**0.0064**	0.77 (0.65–0.91)	**0.0017**	0.79 (0.66–0.94)	**0.0093**
	4	176	0.82 (0.55–1.21)	0.31	1.48 (1.02–2.14)	**0.036**	1.28 (0.88–1.87)	0.19	1.31 (0.86–2)	0.21	0.69 (0.36–1.34)	0.27	0.66 (0.43–1)	**0.046**	1.27 (0.84–1.92)	0.26
Grade	1+2	380	1.28 (0.95–1.73)	0.1	1.15 (0.85–1.56)	0.37	0.59 (0.44–0.79)	**0.0004**	0.8 (0.58–1.1)	0.17	0.61 (0.39–0.94)	**0.024**	0.62 (0.46–0.83)	**0.0011**	0.76 (0.57–1.02)	0.064
	3	1015	1.18 (0.99–1.41)	0.072	1.1 (0.92–1.33)	0.3	0.84 (0.7–1)	0.052	1.32 (1.1–1.58)	**0.003**	0.81 (0.61–1.08)	0.16	0.82 (0.69–0.97)	**0.018**	0.73 (0.61–0.88)	**0.0008**
TP53	Mutated	506	1.21 (0.95–1.53)	0.13	1.55 (1.22–1.97)	**0.0003**	1.42 (1.1–1.84)	**0.0072**	1.19 (0.94–1.49)	0.14	0.5 (0.32–0.77)	**0.0015**	1.17 (0.92–1.49)	0.21	1.19 (0.94–1.5)	0.15
	WT	94	1.53 (0.85–2.76)	0.15	0.64 (0.37–1.12)	0.12	0.54 (0.29–0.99)	**0.043**	1.69 (0.95–3.02)	0.072	0.51 (0.16–1.64)	0.25	0.67 (0.38–1.18)	0.17	1.3 (0.72–2.34)	0.38

The bold font indicates the difference was significant statistically. OC, ovarian cancer; OS, overall survival; WT, wild type.

**Table 4:** The relationship between sirtuins and PFS in other different subtypes of OC (Kaplan–Meier plotter).

			SIRT1		SIRT2		SIRT3		SIRT4		SIRT5		SIRT6		SIRT7	
Subtypes		Cases	HR(95% CI)	*P*	HR(95% CI)	*P*	HR(95% CI)	*P*	HR(95% CI)	*P*	HR(95% CI)	*P*	HR(95% CI)	*P*	HR(95% CI)	*P*
Stage	1	96	3.74 (1.17–11.99)	**0.018**	3.17 (1.06–9.52)	**0.03**	4.26 (1.33–13.62)	**0.0077**	2 (0.67–5.97)	0.21	3.11 (0.89–10.8)	0.06	0.43 (0.14–1.31)	0.13	2.85 (0.95–8.51)	**0.0498**
	2	67	2.04 (0.99–4.21)	**0.049**	0.74 (0.36–1.52)	0.41	0.52 (0.24–1.14)	0.096	0.63 (0.31–1.3)	0.21	2.64 (1.03–6.76)	**0.036**	0.6 (0.3–1.21)	0.15	0.63 (0.28–1.41)	0.26
	3	919	0.88 (0.75–1.04)	0.13	1.42 (1.21–1.66)	**1.5e−05**	1.15 (0.99–1.34)	0.069	1.27 (1.09–1.48)	**0.0025**	1.13 (0.89–1.43)	0.3	1.27 (1.08–1.51)	**0.0048**	1.19 (1–1.43)	0.056
	4	162	0.88 (0.59–1.3)	0.52	1.88 (1.27–2.8)	**0.0015**	1.77 (1.21–2.59)	**0.0028**	0.73 (0.5–1.08)	0.11	1.68 (0.98–2.86)	0.056	0.71 (0.48–1.06)	0.096	0.8 (0.55–1.16)	0.24
Grade	1+2	293	1.31 (0.99–1.74)	0.061	1.45 (1.08–1.94)	**0.012**	0.7 (0.51–0.95)	**0.023**	1.57 (1.16–2.12)	**0.0032**	0.73 (0.48–1.09)	0.12	0.78 (0.59–1.04)	0.085	0.79 (0.58–1.1)	0.16
	3	837	0.85 (0.71–1.01)	0.064	1.31 (1.09–1.57)	**0.0039**	1.24 (1.05–1.46)	**0.012**	1.31 (1.11–1.55)	**0.0015**	1.29 (0.99–1.68)	0.063	0.88 (0.73–1.06)	0.17	0.89 (0.74–1.07)	0.22
TP53	mutated	483	1.33 (1.05–1.68)	**0.018**	1.65 (1.32–2.06)	**1.1e−05**	1.53 (1.21–1.94)	**0.00042**	1.17 (0.94–1.47)	0.16	0.75 (0.5–1.11)	0.15	1.43 (1.12–1.82)	0.0037	1.36 (1.05–1.76)	**0.019**
	WT	84	1.84 (1.07–3.18)	**0.026**	1.91 (1.01–3.63)	**0.043**	0.67 (0.36–1.23)	0.19	1.66 (0.97–2.86)	0.063	1.78 (0.65–4.86)	0.26	1.51 (0.86–2.66)	0.15	1.44 (0.85–2.45)	0.17

The bold font indicates the difference was significant statistically. OC, ovarian cancer; PFS, progression-free survival; WT, wild type.

### Genetic Alteration Analysis of SIRTs in Patients With OC

**Figure 5:**
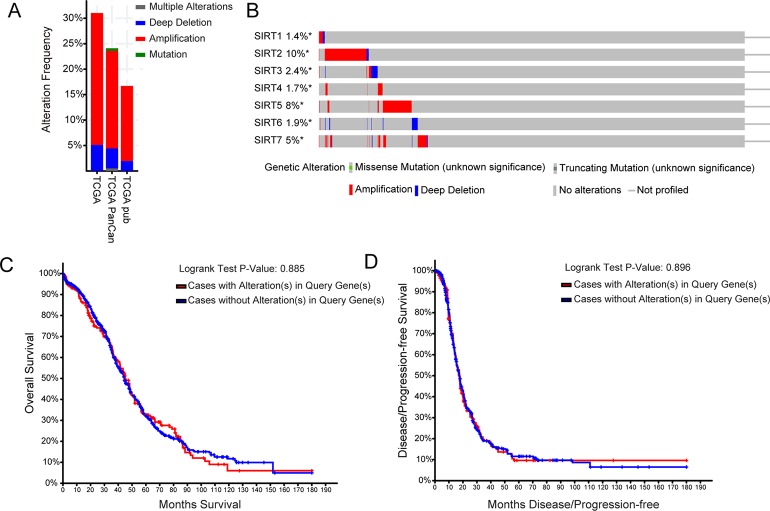
The genetic alteration analysis of SIRTs in patients with OC (cBioPortal). **(A)** Summary of alteration in SIRTs. **(B)** OncoPrint tab summary of alteration on a query of SIRTs. Kaplan–Meier plots comparing **(C)** overall survival (OS) and **(D)** progression-free survival (PFS) in cases with/without *SIRTs* gene alterations.

Next, the genetic alterations of SIRTs in OC patients were explored using the TCGA database and c-BioPortal online tool. SIRTs were altered in 1,754 samples of 1,742 patients from three TCGA databases of serous cystadenocarcinoma, and the alteration rates were 31.02% (188/606), 24.1% (141/585), and 16.7% (94/563), respectively, and the amplification accounted for most changes (**Figure 5A**). As shown in **Figure 5B**, the genetic SIRT alterations occurred in 423 (24%) of the queried samples, and the individual sequence alteration rates varied from 1.4 to 10%. SIRT2, SIRT5, and SIRT7 were ranked as the top 3 of the seven members, and their mutation rates were 10, 8, and 5%, respectively (**Figure 5B**). Using the “Survival” tab with the Kaplan–Meier plot and log-rank test, the survival curves showed that cases with or without alterations in one of the SIRTs had no relationship with OS and PFS (**Figures 5C, D**).

### GO Enrichment and KEGG Pathway Analysis of Protein–Protein Interaction of SIRTs

**Figure 6:**
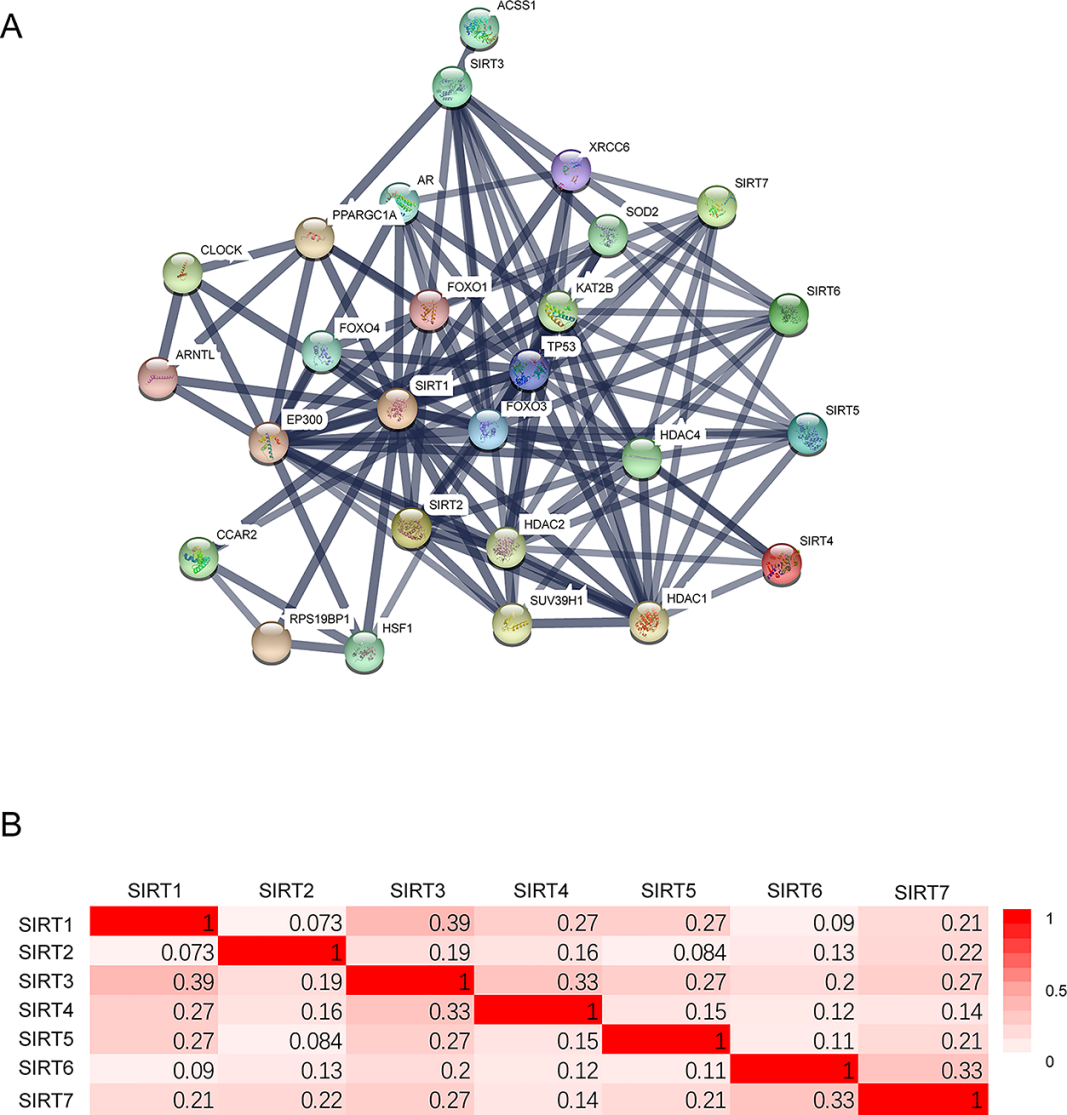
The protein–protein interaction (PPI) of SIRTs. **(A)** The network of 7 SIRT members and 20 proteins that significantly interacted with SIRTs (String). **(B)** The Pearson correlation coefficients between SIRTs (GEPIA).

A network of seven SIRT members and 20 proteins that significantly interacted with SIRTs was constructed using the String database [protein–protein interaction (PPI) enrichment *P* < 1.0E−16]. The network graphic showed that cell metabolism-related genes tumor protein 53 (*TP53*), Fork head box O 1/3/4 (*FOXO1/3/4*), and superoxide dismutase 2 (*SOD2*), and histone posttranscriptional modification-related genes histone deacetylase 1/2/4 (*HDAC 1/2/4*), E1A binding protein p300 (*EP300*), and suppressor of variegation 3–9 homolog 1 (*SUV39H1*) were associated with SIRTs (**Figure 6A**). Then, using “correlation analysis” in GEPIA, the Pearson correlation coefficients were calculated between SIRTs (**Figure 6B**), ranging from 0.073 (SIRT1 vs. SIRT2) to 0.39 (SIRT1 vs. SIRT3).

**Figure 7:**
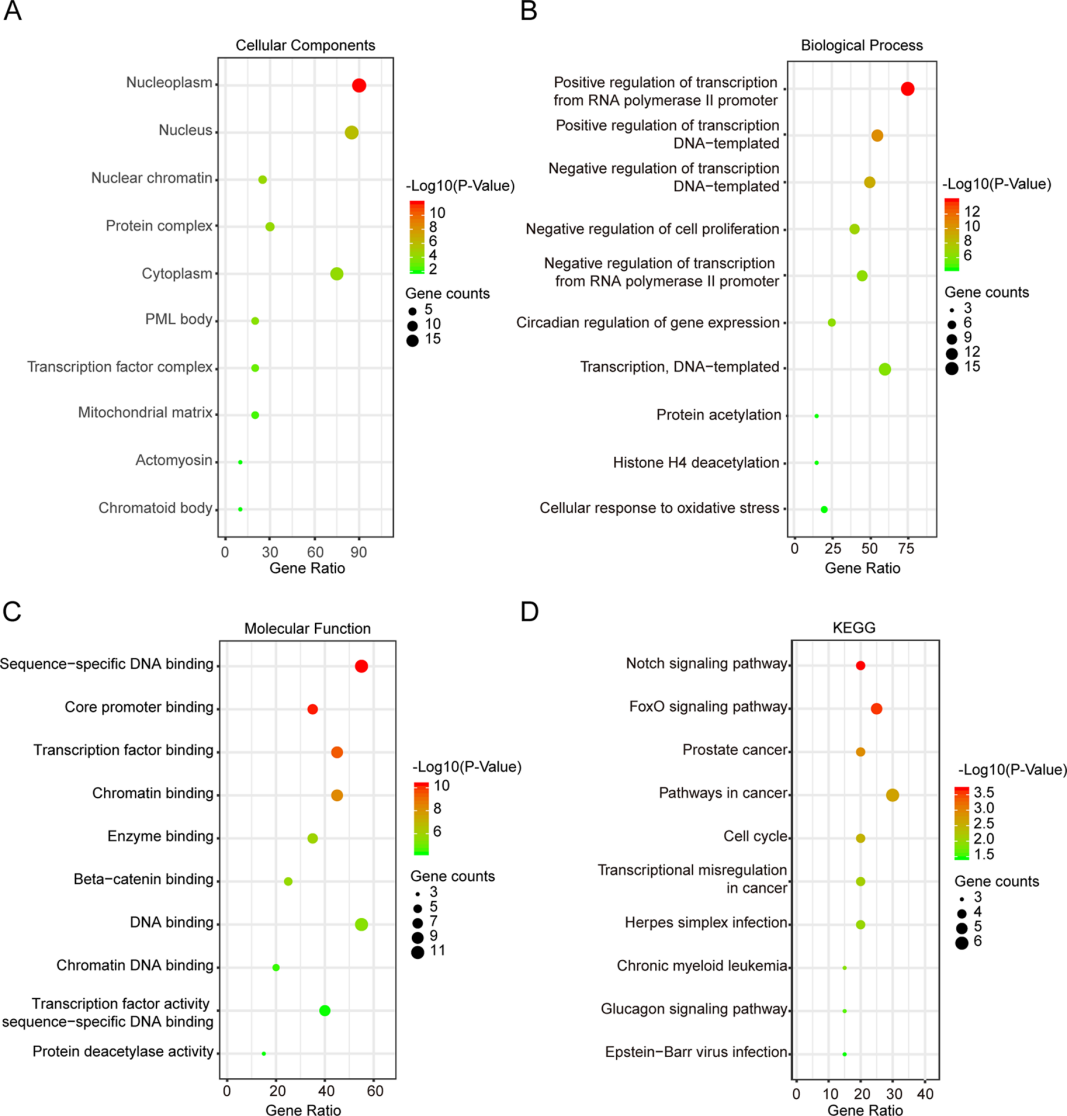
Gene Ontology (GO) enrichment and Kyoto Encyclopedia of Genes and Genomes (KEGG) pathway analysis of SIRTs and their interactors (DAVID). GO enrichment analysis of target genes based on following three aspects: **(A)** cellular component, **(B)** biological process, and **(C)** molecular function. **(D)** KEGG pathway enrichment analysis of target genes.

Next, GO enrichment and KEGG pathway analysis of SIRTs and their interactors were performed using DAVID. Cellular components, biological process, and molecular functions were the three main functions of target host genes in the GO enrichment analysis. The nucleoplasm, nucleus, and cytoplasm were the major cellular components of target genes (**Figure 7A**). Regulation of transcription from RNA polymerase II promoter and DNA templated were mainly associated with SIRTs and their interacting neighbors while binding to DNA, chromatin, and transcription factor were their primary molecular functions predicted online (**Figures 7B, C**). The top 10 KEGG pathways for target genes are shown in **Figure 7D**, and the Notch, FOXO, and cancer pathways were found to be invoved in OC.

## Discussion

Emerging evidence suggest that SIRTs play vital roles in tumorigenesis mediated by their ability to regulate energy metabolism, DNA damage repair, genome stability, and other cancer-associated cellular processes. However, the distinct roles of seven SIRT members in OC are yet to be elucidated. In the current study, the mRNA expression patterns, prognostic values, genetic alterations, and PPI networks of SIRTs in OC patients were investigated through various large databases, including Oncomine and GEPIA, Kaplan–Meier Plotter, cBioPortal, and String. Moreover, GO enrichment and KEGG pathway were also analyzed *via* DAVID.

SIRT1 is the most studied of these seven SIRT members in human cancer and plays dual roles in numerous malignancies including OC (Chalkiadaki and Guarente, 2015). For example, the expression of SIRT1 was significantly higher in endometrioid, mucinous, and clear-cell OC than it was in normal ovaries in IHC analysis, and its overexpression predicted shorter survival in OC (Mvunta et al., 2017). Moreover, overexpression of nuclear SIRT1 was also found to induce chemoresistance and poor prognosis in 63 OC patients (Shuang et al., 2015). Consistently, SIRT1 was found to be involved in the high expression of cancer stem cell markers, chemoresistance, tumorigenesis, and epithelial to mesenchymal transition (EMT) phenotype (Qin et al., 2017). In contrast to these findings, SIRT1 was downregulated in OC based on public datasets and acts as a tumor suppressor (Hyde et al., 2018). In our study, the mRNA expression of SIRT1 was markedly lower in OC tissues than it was in normal tissues. Interestingly, a higher mRNA expression of SIRT1 was significantly associated with poor outcome in OC.

SIRT2 was initially implicated in mitotic progression and serves as a cell cycle regulator (Dryden et al., 2003). Recently, several studies have highlighted the critical roles of SIRT2 in maintaining genome stability (Kim et al., 2011; Serrano et al., 2013), suggesting that this SIRT mainly functions as a tumor suppressor (Chalkiadaki and Guarente, 2015). For example, SIRT2 expression in serous OC was significantly lower than it was in ovarian surface epithelium as determined using Western blotting and IHC. Reduced expression of SIRT2 upregulated cyclin-dependent kinase 4 (CDK4) expression, which eventually accelerated cell proliferation, migration, and invasion, indicating that SIRT2 plays a tumor-suppressor role in OC (Du et al., 2017). Consistently, in the present study, the mRNA expression of SIRT2 was considerably more decreased in OC, especially serous and endometrioid subtypes, than it was in normal tissues and increased levels predicted favorable OS and PFS in patients with OC. However, overexpression of SIRT2 was previously reported to have been related to a poor prognosis in 491 patients with OC (Teng and Zheng, 2017). We assumed that this discrepancy may be due to the high mutation rate of *SIRT2* (10%) in OC, which was identified in our study.

SIRT3 primarily serves as a tumor suppressor by limiting reactive oxygen species levels and antagonizing hypoxia-inducible factor 1-α, which fights against a metabolic switch to aerobic glycolysis (Bell et al., 2011b; Finley et al., 2011; Chalkiadaki and Guarente, 2015). SIRT3 was reported to be downregulated in both metastatic tissues and cell lines of OC and inhibit EMT by interacting with and repressing Twist (Xiang et al., 2016). Moreover, SIRT3 was reported to be activated by S1, a novel pan B-cell lymphoma-2 inhibitor, and then it exerted a proapoptotic effect in SKOV3 OC cells (Dong et al., 2016). SIRT3 was identified to decrease and function as an independent favorable prognostic factor for OS in serous OC (Li et al., 2019). Similarly, our study demonstrated that the transcription levels of SIRT3 in different subtypes of OC were remarkably lower than those in normal samples, and its increased mRNA expression was significantly associated with tumor stage II and favorable outcome in OC. In addition, our results showing that the genetic alteration rate of *SIRT3* was 2.4% and extensive deletion predominately occurred were in line with the findings that at least one copy of the *SIRT3* gene was deleted in 40% of breast cancers and OC, and focal deletions of *SIRT3* were especially frequent (Finley et al., 2011).

SIRT4 has been largely reported to have protective roles against cancer by repressing glutamine metabolism and maintaining genomic stability (Fernandez-marcos and Serrano, 2013; Chalkiadaki and Guarente, 2015). However, its expression pattern and prognostic value in OC have been rarely reported. Only one meta-analysis suggested that lower expression of the *SIRT4* gene was found in a series of solid carcinomas including OC than in corresponding normal tissue (Csibi et al., 2013). Likewise, our results showed that a lower mRNA expression of SIRT4 was found in OC than in normal tissues. Interestingly, a decreased level of SIRT4 was associated with unfavorable OS and PFS in OC, especially in serous subtypes. Although it is not clear, we ascribed the contradictory findings to the background heterogeneity between different databases.

SIRT5 is a unique member of the SIRT family, which possesses multiple enzymatic activities including NAD-dependent histone deacetylase (Nakagawa et al., 2009), potent lysine demalonylase, desuccinylase (Du et al., 2011), and lysine glutarylase (Tan et al., 2014), now known to play controversial roles in tumorigenesis. However, an understanding of the distinct role of SIRT5 in OC is still in its infancy. An analysis of human high-grade serous ovarian carcinomas revealed that the region encompassing the *SIRT5* locus was amplified in 30% of these tumors (Bell et al., 2011a). Consistently, our results showed *SIRT5* gene alteration in 8% of queried OC patients and amplifications accounted for most CNAs. Moreover, SIRT5 was found to increase in primary serous OCs/tubal cancers compared with that in normal tissues, and high expression of it was associated with better OS by univariable analysis (Li et al., 2019). Similarly, in our study, a higher mRNA level of SIRT5 was found in OC, especially in serous adenocarcinoma, and it was related to poor PFS in OC. Interestingly, increased expression of SIRT5 predicted superior OS, and this may be partly due to its marked overexpression in early tumor stages.

SIRT6 and SIRT7 are both nuclear proteins with deacetylase activity and function as both tumor suppressor and promotor in cancer, including OC (Chen et al., 2013; Chalkiadaki and Guarente, 2015). The mRNA expression of SIRT6 in 32 OC tissue samples was remarkably lower than that in the paired normal tissues, and SIRT6 inhibited the proliferation of OC cells by suppressing Notch 3 expression (Zhang et al., 2015). Conversely, the expression of SIRT6 was associated with higher tumor stage, higher histological grade, platinum resistance, and predicted shorter OS in 104 patients with OC. Moreover, SIRT6 was overexpressed in omental metastases compared with corresponding primary counterparts (Li et al., 2019) and facilitated the invasiveness of OC cells by regulating EMT signaling, but it did not inhibit their proliferation (Bae et al., 2018).

SIRT7 was overexpressed in OC tissues and cell lines (Barber et al., 2013), omental metastasis tissues (Li et al., 2019), and promoted tumor cell proliferative potential *via* regulating apoptosis (Wang et al., 2015). However, SIRT7 was significantly reduced in cultured chemoresistant OC cells (Aljada et al., 2014) and was considered a tumor suppressor based on its inhibition of the activity of HIF-1 and HIF-2 transcription factors (Hubbi et al., 2013). The present study demonstrated that SIRT6 and SIRT7 levels were slightly lower in OC than normal conditions based on the GEPIA database analysis (*P* > 0.05) but significantly upregulated in the Oncomine database. Moreover, overexpression of SIRT6 and SIRT7 was associated with tumor stage II and a better outcome.

In addition to the individual prognostic values of the investigated SIRTs, we further determined the simultaneous increase in the mRNA expression of all SIRTs predicted poor prognosis and whether the genes altered or not had no relationship with OS and PFS. In addition, the enrichment analysis indicated that SIRTs and their 20 interactors were mainly correlated with cancer-related pathways such as the Notch and FOXO pathways.

Despite the numerous findings, there are some limitations to this study. First, this was a bioinformatics analysis mainly based on transcriptional data, whereas proteins are the primary mediators of the various functions. Moreover, although SIRTs showed distinct prognostic values in OC, the multivariable analyses of molecules such as breast cancer type 1, human epididymis protein 4, and cancer antigen 125 are needed for further identification. Thus, the utility of SIRT expression as independent prognostic indicators in OC is yet to be further confirmed. Finally, since all the data were obtained from different databases with inevitable background heterogeneity, our results may contain some inconsistency. To address these issues, we are planning to perform well designed studies to verify these findings in the near future.

In conclusion, the mRNA expression patterns, prognostic values, genetic alterations, and PPI networks of SIRTs in OC patients were investigated. This comprehensive bioinformatics analysis revealed that SIRT1–4, 6, and 7 may be new prognostic biomarkers, and SIRT5 may be a potential target for precision therapy for patients with OC. However, further studies are needed to confirm this notion. Finally, these findings would contribute to a better understanding of the distinct roles of SIRTs in OC.

## Data Availability

Publicly available datasets were analyzed in this study. This data can be found here: www.oncomine.org, http://gepia.cancer-pku.cn/, http://kmplot.com/analysis/, https://www.cbioportal.org/, https://string-db.org/, https://david.ncifcrf.gov/.

## Ethics Statement

The studies involving human participants were reviewed and approved by Medical Research Ethics Committee of China Medical University. The patients/participants provided their written informed consent to participate in this study.

## Author Contributions

All authors contributed to study design, data analysis, drafting, or revising the article, gave final approval of the version to be published, and agree to be accountable for all aspects of the work.

## Funding

This work was supported by the National Natural Science Foundation of China (grant numbers 81672964, 81874214, and 81702269).

## Conflict of Interest Statement

The authors declare that the research was conducted in the absence of any commercial or financial relationships that could be construed as a potential conflict of interest.
